# Evaluation of errors related to surgical pathology specimens of different hospital departments with a patient safety approach: a case study in Iran

**DOI:** 10.1186/s13037-023-00360-1

**Published:** 2023-04-18

**Authors:** Seyed Saeed Tabatabaee, Vahid Ghavami, Rohollah Kalhor, Mohammad Amerzadeh, Hadi Zomorrodi-Niat

**Affiliations:** 1 Social Determinants of Health Research Center, Mashhad University of Medical Sciences, Mashhad, Iran; 2 Department of Health Economics and Management Sciences, School of Health, Mashhad University of Medical Sciences, Mashhad, Iran; 3 Department of Biostatistics, School of Health, Mashhad University of Medical Sciences, Mashhad, Iran; 4 Social Determinants of Health Research Center, Research Institute for Prevention of Non-Communicable Diseases, Qazvin University of Medical Sciences, Qazvin, Iran; 5 Student Research Committee, Mashhad University of Medical Sciences, Mashhad, Iran

**Keywords:** Patient safety, Labeling error, Pathology specimen, Hospital, Preanalytical error

## Abstract

**Background:**

Most surgical specimen errors occur in the pre-analysis stage, which can be prevented. This study aims to identify errors related to surgical pathology specimens in one of the most comprehensive healthcare centers in Northeast Iran.

**Methods:**

The present study is descriptive and analytical research conducted cross-sectionally in 2021 at Ghaem healthcare center in the Mashhad University of Medical Sciences on the basis of a census sampling. We used a standard checklist to collect information. Professors and pathologists evaluated the validity and reliability of the checklist using Cronbach’s alpha calculation method of 0.89. We analyzed the results using statistical indices, SPSS 21 software, and the chi-square test.

**Results:**

Out of 5617 pathology specimens studied, we detected 646 errors. The highest number of errors is the mismatch of the specimen with the label (219 cases; 3.9%) and the non-compliance of the patient’s profile in the specimen sent with the label (129 cases; 2.3%), and the lowest errors are the inappropriate volume of the fixator(24 cases; 0.4%), and they accounted for insufficient sample size (25 cases; 0.4%). Based on Fisher’s exact test results, there was a significant difference between the proportion of errors in different departments and months.

**Conclusion:**

Considering the frequency of labeling errors in the stage before the analysis in the pathology department, the use of barcode imprinted in specimen containers, the removal of the paper request for pathology, the use of radio frequency chip technology, the use of the rechecking system and improving communication in different departments can be effective in reducing these errors.

## Background

Patient safety is a strategic priority for senior managers of the health system. Managers should pay special attention to the evaluation of safety culture in healthcare organizations, especially hospitals to promote patient safety [1]. The patient safety culture in the hospital is inappropriate and requires urgent intervention [2]. Promoting patient safety culture can effectively reduce the medical errors and address concerns related to the lack of safety in health systems by recognizing the factors causing errors [2, 3].

Surgical specimen collection is a routine process occuring daily in hospitals [4], and various errors can occur in each of the three stages of the specimen management process. They are pre-analysis (from the operating room to the pathology), analysis (from receiving in pathology to analysis), and post-analysis (from analysis to reporting results) [5].

One primary concern of patient safety is the errors related to managing surgical specimens, especially in the pre-analysis stage [5]. Most surgical specimen errors happen in the pre-analysis stage, which can be prevented [6]. Misuse surgical specimens increases the risk of preventable harm through delayed treatment, incorrect treatment selection, or misdiagnosis [4].

The errors that are attributed to the pre-analytical phase are mismatch of specimen form and request [4, 6–9], incorrect patient identification [6–8, 10], unlabeled specimens [4, 6–8]; Incorrect number of specimens [6], incorrect order entry [7], incorrect specimen identification [7], incorrect laterality on specimen label [6, 10], incorrect specimen in the container [7], missing specimen(s) [11], incorrect fixator or holder [11] and delay in transporting to incorrect destination, and incorrect method of transporting [11].

The problem areas analysis shows that patient identification is a crucial issue. Most errors occur in the labeling of test tubes (45.4%) and analysis forms or request sheets (33%) [12]. Mislabeling laboratory specimens creates problems in the overall process of laboratory medicine diagnosis and can cause fatal patient harm [12]. Therefore, the ISO standard specifies the need to evaluate, monitor, and improve all procedures and processes in the pre-analysis phase, which includes the test request phase and specimen collection [13]. Therefore, identifying errors and reducing them in the pre-analytical stage is necessary to ensure cost-effectiveness, patient satisfaction, and high-quality laboratory services [14].

Analyzing these error reports and the complexity of the specimen collection process poses significant challenges for healthcare professionals. Although the rate of these errors can be used as one index of patient safety for patients undergoing surgery, the rate of these errors has been studied very little. This study aims to identify errors related to pathology specimens in the Ghaem healthcare center at the Mashhad University of Medical Sciences.

### **Methods**

The current study is descriptive and analytical research conducted cross-sectionally in 2021 in one of the largest healthcare centers in the country’s northeast. The information sources of this study included all the pathology request forms completed in different hospitals departments and sent to the pathology laboratory for the diagnostic process. In this study, sampling was not done, and the study was done on the basis of a census sampling.

We identified errors related to pathology specimens from the difference between the information recorded in the pathology request form and the information on the specimen label sent to the pathology laboratory. For this purpose, after obtaining the permission to conduct the research, we sent the checklist for identifying the errors of the pathology specimens to the pathology unit in Ghaem healthcare center and collected the information and analyzed in six months. We classified errors based on error type and specimen location. We identified the type of error through any inconsistency between the information recorded on the application form and the specimen label. For example, the error of the unlabeled specimen refers to the unlabeled specimens, the wrong side error is paired specimens such as eyes and ears, and the errors related to the location of the specimen include the location of the tissue, which includes breast, skin, etc.

We used Makary’s standard checklist to collect information [15]. Face and content validity were used to measure the validity of the checklist. We provided the initial checklist to 5 professors and pathologists to reach a consensus on the checklist (face validity). We also used the content validity index and CVR calculation to measure content validity. For this purpose, a checklist with a measurement format was designed and completed with 30 experts’ opinions. The retest method was used to measure the reliability of the checklist. We analyzed the results using statistical indices, descriptive statistics, and Fisher’s exact test using SPSS software version 21.

## **Results**

We studied 5617 pathology specimens, of which 4971 (88.5%) specimens did not have any errors, and the error of “non-matching of the specimen with the label.

” with 219 (3.9%) had the highest number of errors. The details of the distribution of error types are reported in Table 1.

**Table 1:** Distribution of errors in pathology specimens

Type of error	Number	Percentage
Without error	4971	88.5
Not-matching of the specimen with the label	219	3.9
Not-compliance of the patient’s profile in the specimen sent with the label	129	2.3
Not-mentioning the full demographic information of the patient on the label or incorrect information	93	1.7
Not-mentioning the specifications of the technician completing the label	76	1.4
Not mentioning the patent history	51	0.9
Not mentioning the location of the specimen	29	0.5
Insufficient specimen	25	0.4
Inappropriate volume of fixator	24	0.4
Total	5617	100.0

Among the pathology specimens sent from different departments, the highest error ratio was related to surgical departments with 17.6% and the lowest was related to operating rooms with 9.9% (Table 2).

**Table 2:** The status of pathology errors by different department

The department	Without errors	Percentage	Error	Percentage	Total	Percentage
Operating rooms	2872	90.12%	315	9.88%	3187	100%
Surgical departments	530	82.43%	113	17.57%	643	100%
Maternity	749	86.89%	113	13.11%	862	100%
Non-surgical	397	88.81%	50	11.19%	447	100%
Endoscopy	210	87.50%	30	12.50%	240	100%
Intensive Care Units	213	89.50%	25	10.50%	238	100%
Total	4971	88.50%	646	11.50%	5617	100%

Based on Fisher’s exact test, the percentage of errors in different sections was significantly different (p < 0.001).

Regarding the status of errors in different months, we observed the highest percentage of errors in the first quarter (Table 3). Also, based on Fisher’s exact test, a significant relationship between month and error rate was observed (P < 0.001).

**Table 3:** The pathology errors in different months

Months	Without error	Percentage without error	Error	Percentage error	Total	Percentage
April	775	84.61%	141	15.39%	916	100%
May	847	85.73%	141	14.27%	988	100%
June	1081	84.12%	204	15.88%	1285	100%
July	1165	91.37%	110	8.63%	1275	100%
August	500	95.79%	22	4.21%	522	100%
September	603	95.56%	28	4.44%	631	100%
Total	4971	88.50	646	11.50%	5617	100%

Regarding the status of errors by insurance, the highest percentage of errors was related to the uninsured, and the lowest was related to other insurances (Table 4). Based on Fisher’s exact test, the percentage of pathology error was significantly different in different types of insurance (p = 0.005).

**Table 4:** Pathology errors according to insurance types

Type of insurance	without error	Percentage without error	Error	Percentage of total error	Total	Percentage
The uninsured	152	81.28%	35	18.72%	187	100%
Health insurance	3125	88.08%	423	11.92%	3548	100%
Social Security Insurance	1504	89.79%	171	10.21%	1675	100%
Other insurances	190	91.79%	17	8.21%	207	100%
Total	4971	88.50%	646	11.50%	5617	100%

## **Discussion**

This study aimed to identify errors related to pathology specimens in one of the largest healthcare centers in Iran northeast. This study focused on the pre-analysis stage (transferring information from the doctor to the nurse, labeling, packaging, and transferring the specimen). Since it has been less investigated in other studies and due to the lack of supervision in this stage, differences in the way specimens are labeled and transported can be a valuable source of patient harm.

The overall labeling errors in the studied hospital is 11.5%. On average, for every 1000 pathology specimens sent from different departments of the hospital, 115 errors occur, and this number of errors raises significant risks for the patient safety. Jesica et al. identified 234 errors (6.8%) in a review of 33,962 pathology specimens, ten errors per 1000 specimens [16]. A review of 8288 pathology specimens identified 5.8% errors per 1000 specimens [17]. Bülbüloğlu et al. identified a 0.32% specimen error in a 21,078 pathology specimens [18]. Nakhleh and Zarbo, in a review of over one million surgical pathology specimens from 417 institutions around the world, concluded that the error in the identification of pathology specimens occurs in 6% of the specimens [19]. Makary et al. identified 4.3 errors per 1000 examined specimens [15]. The present study’s increase in pathology errors compared to similar studies is due to the lack of error prevention methods in the studied hospital.

The results showed that the error of non-matching the specimen with the label 219 (3.9%) has the highest error rate among the errors. After that, the highest error is related to the non-compliance of the patient’s profile in the specimen with the label at 129 (2.3%). In a study by Syndman et al., after extensive analysis of laboratory reports in 30 healthcare organizations, they found that laboratory events before analysis were the most common (81%) errors. The top three examples of errors were unlabeled specimens (18.7%), wrongly labeled specimens (16.3%), and improper collection (13.2%) [20]. In the study of Makary et al., in the evaluation of 21,351 surgical specimens, 11 cases (0.05%) of inappropriate label errors were discovered, which led to assigning the specimen to the wrong patient [15]. Francis et al. examined over 8000 containers containing pathology specimens and identified 0.09% wrong labels [21]. In Tabatabai et al.’s study, the most common errors were non-recording the patient’s age (9%), not registering the patient’s father name (9%), and not recording the number of biopsy specimens (9%) [22]. Its difference with this study is in the type of data collection form. The lack of continuous training of personnel and the lack of sufficient supervision caused this type of error to be high in the studied hospital. The results showed that using two patient identification codes simultaneously reduced such errors [8]. Therefore, the simultaneous use of two identification codes for patients can significantly reduce the errors related to not registering the identification code or incorrect registering the identification code of the patients.

The status of department errors showed that even though most cases of pathology sampling were in operating rooms, the lowest detected error was in operating rooms at 9.9%. Despite the third place in the number of specimens taken, the surgery department had the highest number of errors with 17.57%. The reason for this could be the greater sensitivity (ability or training) of operating room personnel for specimen preparation. There are ways to reduce errors. The writing technique is rereading that the nurse can use to communicate with the doctor during surgery [5]. The results of one study showed that the use of a pathology specimen management protocol in the operating room reduced the rate of adverse events from 0.3226% (68 of 21,078) to 0.032% (6 of 18,706) after the protocol systematized the surgical pathology specimen management process [18]. The pathology department can quickly identify mislabeled specimens using a simple screening process. This quality control method is a meager cost and has a high acceptance rate, which makes patient safety more effective. Regarding the status of errors by insurance, the highest percentage was related to the uninsured, and the lowest was related to other insurances. This shows that the departments’ staff have been more sensitive to sampling and preventing labeling errors due to the careful monitoring of insurance experts to confirm patients’ files with insurance.

## Conclusion

The quality control without accepting the possibility of error is impossible. Incorrect labeling of pathology specimens is a significant source of medical errors cause harm to the patient. The percentage of labeling errors in the pre-analysis stage in the pathology department of the studied healthcare center was high, which necessitates the use of preventive risk assessment strategies to identify weak points. Investing in continuous training of employees with an emphasis on patient safety, improvement initiatives such as simplifying processes, using barcode technology in specimen containers, eliminating paper pathology request forms, using radio frequency chip technology, using recheck systems, and improving communication in operating rooms, such as using a surgical checklist to increase team communication and improve team culture, can reduce the number of errors in labeling surgical specimens.

### Rigor of study

This is the first kind of study conducted at Mashhad University of Medical Sciences. Nonetheless, we had some limitations. The most important one was conducting a study in one hospital. We also did not include private hospitals in our study.

## Data Availability

The datasets used and/or analyzed during the current study available from the corresponding author on reasonable request. The entire dataset is in Farsi language. The Data can be available in English language for the readers and make available from the corresponding author on reasonable request.
